# Thirty Years of Orphan Drug Legislation and the Development of Drugs to Treat Rare Seizure Conditions: A Cross Sectional Analysis

**DOI:** 10.1371/journal.pone.0161660

**Published:** 2016-08-24

**Authors:** Jan Henje Döring, Anette Lampert, Georg F. Hoffmann, Markus Ries

**Affiliations:** 1 Pediatric Neurology and Metabolic Medicine, Center for Pediatrics and Adolescent Medicine, Heidelberg University Hospital, Im Neuenheimer Feld 430, 69120 Heidelberg, Germany; 2 Center for Rare Diseases, Heidelberg University Hospital, Im Neuenheimer Feld 430, 69120 Heidelberg, Germany; 3 Department of Clinical Pharmacology and Pharmacoepidemiology, Heidelberg University Hospital, Im Neuenheimer Feld 410, 69120 Heidelberg, Germany; 4 Cooperation Unit Clinical Pharmacy, Heidelberg University Hospital, Im Neuenheimer Feld 410, 69120 Heidelberg, Germany; University of Catanzaro, ITALY

## Abstract

**Background:**

Epilepsy is a serious chronic health condition with a high morbidity impairing the life of patients and afflicted families. Many epileptic conditions, especially those affecting children, are rare disorders generating an urgent medical need for more efficacious therapy options. Therefore, we assessed the output of the US and European orphan drug legislations.

**Methods:**

Quantitative analysis of the FDA and EMA databases for orphan drug designations according to STrengthening the Reporting of OBservational studies in Epidemiology (STROBE) criteria.

**Results:**

Within the US Orphan Drug Act 40 designations were granted delivering nine approvals, i.e. clobazam, diazepam viscous solution for rectal administration, felbamate, fosphenytoin, lamotrigine, repository corticotropin, rufinamide, topiramate, and vigabatrin. Since 2000 the EMA granted six orphan drug designations whereof two compounds were approved, i.e. rufinamide and stiripentol. In the US, two orphan drug designations were withdrawn. Orphan drugs were approved for conditions including Lennox-Gastaut syndrome, infantile spasms, Dravet syndrome, and status epilepticus. Comparing time to approval for rufinamide, which was approved in the US and the EU to treat rare seizure conditions, the process seems faster in the EU (2.2 years) than in the US (4.3 years).

**Conclusion:**

Orphan drug development in the US and in the EU delivered only few molecular entities to treat rare seizure disorders. The development programs focused on already approved antiepileptic drugs or alternative pharmaceutical formulations. Most orphan drugs approved in the US are not approved in the EU to treat rare seizures although some were introduced after 2000 when the EU adopted the Orphan Drug Regulation.

## Introduction

Epilepsy is a serious chronic health condition with a high morbidity impairing the life of patients and affected families through seizures, hospitalizations, emergency department visits, and medication burden [1, 2]. Particularly, seizure onset in childhood can compromise the child’s development and frequently causes lifelong disability and dependency [2]. Epilepsy comprises a large group of syndromes whereof some meet the criteria for a rare disease according to the World Health Organization (WHO), i.e. a condition affecting less than 65–100 in 100,000 inhabitants [3]. For example, Lennox-Gastaut syndrome with an estimated prevalence of 15/100,000, West syndrome (infantile spasms) with an estimated prevalence of 8/100,000, Dravet syndrome (severe myoclonic epilepsy in infancy) with an estimated birth-prevalence of 2.5/100,000, or Pyridoxine-dependent epilepsy with 0,2/100,000 fulfill the WHO definition of a rare disease [4]. Today clinically available anti-epileptic drugs can control seizures in approximately two-third of patients [5–7], particularly in rare seizure conditions such as Lennox-Gastaut or Dravet syndrome long term prognosis is guarded and most patients are refractory to medical treatment [8, 9]. Psychomotor delay and neuropsychiatric symptoms occur regularly. In addition, most often anti-epileptic pharmacotherapy is limited by drug-drug interactions, adverse drug events, and complex dose regimens impairing adherence [10–13].

Since 1983, the US Orphan Drug Act has stimulated the development of orphan drugs by granting various incentives, such as seven years marketing exclusivity, tax credit for 50% of clinical trial costs, protocol assistance, Food and Drug Administration (FDA) fee waiver, and orphan products grant programs [14]. A compound qualifies for the incentives described in the US Orphan Drug Act when a disease affects less than 200,000 patients in the US or when economic viability is lacking although prevalence exceeds 200,000 [3]. In 1999, the European Medicines Agency (EMA) adopted the legislation for orphan drugs (Regulation (EC) No 141/2000), which came into force in 2000, to stimulate orphan drug development in the European Union (EU) by granting, for example, up to ten years marketing exclusivity after approval (plus two years for orphan drugs with a pediatric investigation plan), fee reduction, and protocol assistance [15]. In the EU, a compound qualifies for orphan drug designation when it is indicated for a life-threatening or chronically debilitating condition affecting less than five in 10,000 persons or when it is improbable to sufficiently generate return of investment although a life-threatening, seriously debilitating or serious and chronic condition affects more than five in 10,000 patients [16]. A furtherprerequisite is absence of a satisfactory method of diagnosis, prevention, or treatment, or if it exists, the new medicinal product must be of significant benefit to the patients [16].

During the last decades scientists, policy makers, and pharmaceutical companies have advocated to respond to challenges in orphan drug development. In addition, political and legislative developments, such as the US Orphan Drug Act and the Orphan Drug Regulation in the EU, have changed the environment in orphan drug development. Considering the unmet medical need for anti-epileptic treatments, drug development in orphan epilepsy—as in any rare disease—is challenged by small sample sizes, heterogeneous pathomechanisms, and involvement of children. Therefore, we systematically analyzed the impact of the US Orphan Drug Act and the Orphan Drug Regulation in the EU on orphan drug development in rare seizure conditions by investigating orphan drug designations and approvals, time to approval, compounds and indications. In addition, we examined pivotal trial designs to illustrate quality indicators, such as randomization or control, in clinical research of approved orphan drugs to treat rare seizure conditions.

## Methods

This cross-sectional analysis was conducted according to STrengthening the Reporting of OBservational studies in Epidemiology (STROBE) criteria.

### Data acquisition

In December 2015, we searched the FDA database “Search Orphan Drug Designations and Approvals” [17], the EMA databases “Rare disease (orphan) designations” [18], “European public assessment reports/orphan medicines” [19], and “Register of designated Orphan Medicinal Products” [20] for designated and approved orphan drugs to treat rare seizure conditions. First a semantic search was performed using search terms, such as seizure, epilepsy, status epilepticus, and spasm followed by a specific search for epilepsy syndromes based on the ILAE definition of electroclinical syndromes and other epilepsies [21]: West syndrome, Dravet syndrome, myoclonic encephalopathy, Panayiotopoulos syndrome, Lennox-Gastaut syndrome, Landau-Kleffner syndrome, Otahara syndrome, and early myoclonic encephalopathy. Additionally, an inverse search for known and new substances for epilepsy and seizure treatment based on recent EILAT reports [22] and ATC code (N03A Antiepileptics) was added: brivaracetam, bumetanide, cannabidiol, cannabidivarin, carbamazepine, carisbamate, clobazam, clonazepam, diazepam, divalproex, eslicarbazepine, ethosuximide, ezogabine or retigabine, felbamate, fosphenytoin, gabapentin, ganaxolone, lacosamide, lamotrigine, levetiracetam, lorazepam, metharbital, oxcarbazepine, paramethadione, perampanel, phenacemide, phenobarbital, phenytoin, phensuximide, pregabalin, primidone, rufinamide, stiripentol, tiagabine, topiramate, trimethadione, valproic acid or valproate, vigabatrin, and zonisamide. The Orphanet Report series was consulted for epidemiological data on rare seizure conditions [4]. Information on design and endpoints of clinical trials was extracted from the drug label which was accessed by entering the respective drug name as search term at https://www.accessdata.fda.gov/scripts/cder/drugsatfda/ or from the European Public Assessment Reports (EPAR). JHD and AL independently performed the database search and extracted the information from the databases.

### Definitions

Time to FDA approval or marketing authorization by the EMA and European Commission was defined as the time period from orphan drug designation until approval by the FDA or EMA and European Commission.

### Statistical analysis

Data were summarized using techniques of descriptive statistics. As such, continuous variables were summarized with means and standard deviations, and categorical variables were summarized with frequencies and percentages. Statistical analyses were performed using SAS Enterprise Guide version 9.1 (SAS, Cary, NC, USA). Data from the FDA and EMA were analyzed both separately and comparatively. Missing data were not imputed and sensitivity analysis was not performed.

## Results

### Designations and approvals

**Fig 1:**
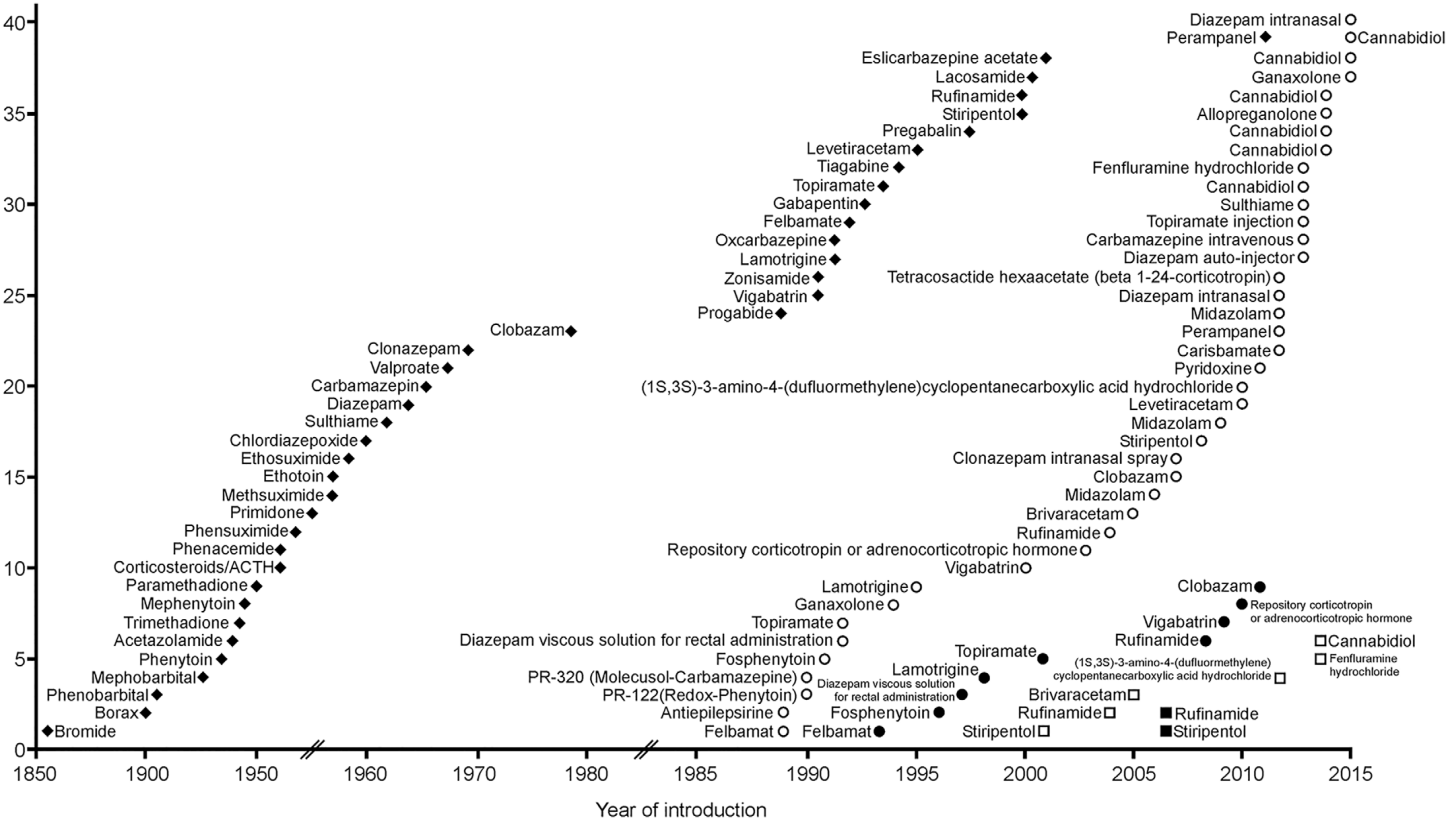
Cumulative number of approved non-orphan antiepileptic drugs (◆) illustrating the year of first licensing or the first mention of clinical use in a country of Europe, the United States, or Japan (adapted from [31]). Cumulative number of US orphan drug designations (○) and approvals (●). Cumulative number of orphan drug designations (□) and approvals (■) in the EU.

The FDA granted 40 orphan drug designations for treatment of rare seizure conditions resulting in nine approvals representing an acceptance rate of 23% (Fig 1). Two designations, i.e. Pr-122 (redox-phenytoin) and Pr-320 (molecusol-carbamazepine), were withdrawn. Reasons for non-approval were not publicly available. In the EU, six compounds received a positive opinion by the EMA’s Committee on Orphan Medicines (COMP) and were designated as orphan drugs (Fig 1). Midazolam hydrochloride for oromucosal use for the treatment of seizures which continue for at least five minutes received a negative opinion. The European Commission granted a central marketing authorization for two compounds to treat rare seizure conditions, i.e. rufinamide and stiripentol, representing an acceptance rate of 33%. Only rufinamide was designated and approved to treat Lennox-Gastaut syndrome in the US and in the EU. Eight compounds that were approved in the US for treatment of rare seizure conditions and epilepsy syndromes were not submitted for orphan drug designation in the EU, i.e. clobazam, diazepam viscous solution for rectal administration, felbamate, fosphenytoin, lamotrigine, repository corticotropin or adrenocorticotropic hormone, topiramate, and vigabatrin. Three of these compounds (i.e. clobazam, repository corticotropin or adrenocorticotropic hormone, and vigabatrin) were designated and approved in the US after the year 2000 when the Orphan Drug Regulation was already introduced in the EU. In total, 20 designations were obtained in the US after the Orphan Drug Regulation was introduced in 2000 in the EU, while these designations were not obtained in the EU. Two compounds, i.e. fosphenytoin and repository corticotropin or adrenocorticotropic hormone, were first approved within the US Orphan Drug Act (Fig 1).

Until December 2015, the US Orphan Drug Act has delivered 500 approved orphan drugs in total, while in the EU 103 orphan drugs have received marketing authorization since 2000.

### Time to approval

**Fig 2:**
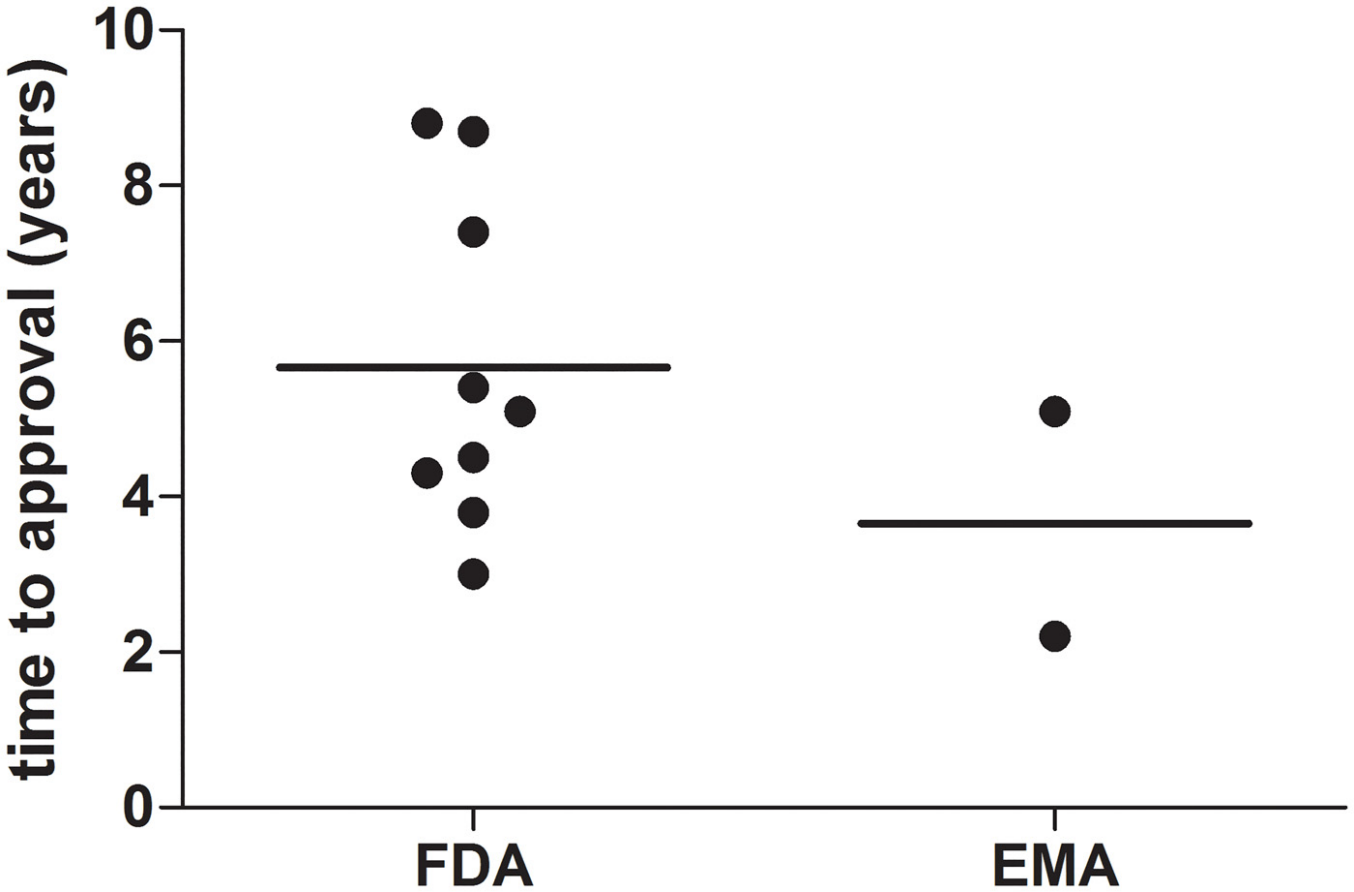
Time to approval of compounds intended to treat orphan epileptic conditions. Lines indicate means.

Mean time to approval for orphan drugs for treating rare seizure conditions was 5.7 years (standard deviation ± 2.0 years, range 3 to 8.8 years) in the US and in the EU 2.2 years for rufinamide and 5.1 years for stiripentol (Fig 2). For rufinamide, a compound that was approved in the US and the EU for the same indication, time to approval was 4.3 years in the US and 2.2 years in the EU.

### Compounds and indications

Cannabidiol obtained most designations (FDA N = 6 and EMA N = 1) (Table 1). Cannabidiol obtained two orphan drug designations by the FDA for treatment of Dravet syndrome (time difference between designations was 2 months) and two orphan drug designations for treatment of Lennox-Gastaut syndrome (time difference between designations was 4 months) (Table 1). Designations within the same conditions were granted by different companies. Designations were most frequently granted for treatment of Lennox-Gastaut syndrome (FDA N = 8 and EMA N = 1), infantile spasms (West syndrome) (FDA N = 7 and EMA N = 1), and Dravet syndrome (severe myoclonic epilepsy in infancy) (FDA N = 4 and EMA N = 3) (Table 1). Orphan drugs were approved for conditions including Lennox-Gastaut syndrome, infantile spasms, Dravet syndrome, and status epilepticus. Most compounds were approved for treatment of Lennox-Gastaut (FDA N = 5 and EMA N = 1) (Table 1). Compounds designated in the US and the EU obtained orphan drug designation for the same indication. Rufinamide was approved in the US and in the EU for treatment of Lennox-Gastaut syndrome. Seven FDA orphan drug designations focused on a route of administration other than the oral route for acute clinical conditions or when patients are unable to take anything by mouth, i.e. intravenous carbamazepine (N = 1), intranasal clonazepam (N = 1), intranasal diazepam (N = 2), diazepam in an auto-injector (N = 1), viscous solution for rectal administration of diazepam (N = 1), and injectable topiramate (N = 1).

**Table 1:** Compounds for rare seizure conditions designated or approved by the FDA and EMA.

Compound	Regulatory authority	Condition	Date of designation (FDA) or positive opinion (EMA)	Date of approval (FDA) or marketing authorization (EMA)
(1S,3S)-3-amino-4-(difluoromethylene) cyclopentanecarboxylic acid hydrochloride	FDA	Treatment of infantile spasms	15 September 2010	n/a
	EMA	Treatment of infantile spasms	09 February 2012	n/a
Allopregnanolone	FDA	Treatment of status epilepticus	20 April 2014	n/a
Antiepilepsirine	FDA	Treatment of drug resistant generalized tonic-clonic epilepsy in children and adults	23 March 1989	n/a
Brivaracetam	FDA	Treatment of symptomatic myoclonus	10 May 2005	n/a
	EMA	Treatment of progressive myoclonic epilepsies	26 August 2005	n/a
Cannabidiol	FDA	Treatment of infantile spasms	23 July 2015	n/a
		Treatment of neonatal hypoxic ischemic encephalopathy	22 April 2015	n/a
		Treatment of Lennox-Gastaut syndrome	23 June 2014	n/a
		Treatment of Lennox-Gastaut syndrome	27 February 2014	n/a
		Treatment of Dravet syndrome	7 January 2014	n/a
		Treatment of Dravet syndrome	14 November 2013	n/a
	EMA	Treatment of Dravet syndrome	15 October 2014	n/a
Carbamazepine intravenous	FDA	Treatment of epilepsy patients who cannot take anything by mouth	27 June 2013	n/a
Carisbamate	FDA	Treatment of infantile spasms	16 March 2012	n/a
Clobazam	FDA	Treatment of Lennox-Gastaut Syndrome	18 December 2007	21 October 2011
Clonazepam intranasal	FDA	Treatment of recurrent acute repetitive seizures	19 December 2007	n/a
Diazepam intranasal	FDA	Management of acute repetitive seizures	16 November 2015	n/a
		Management of acute repetitive seizures	31 July 2012	n/a
Diazepam auto-injector	FDA	Management of selected, refractory patients with epilepsy on stable regimens of antiepileptic drugs, who require intermittent use of diazepam to control bouts of increased seizure activity	30 May 2013	n/a
Diazepam viscous solution for rectal administration	FDA	Management of selected, refractory, patients with epilepsy on stable regimens of antiepileptic drugs, who require intermittent use of diazepam to control bouts of increased seizure activity	25 February 1992	27 July 1997
Felbamate	FDA	Treatment of Lennox-Gastaut syndrome.	24 January 1989	29 July 1993
Fenfluramine hydrochloride	FDA	Treatment of Dravet Syndrome	20 December 2013	n/a
	EMA	Treatment of Dravet syndrome	16 January 2014	n/a
Fosphenytoin	FDA	For the acute treatment of patients with status epilepticus of the grand mal type	06 April 1991	08 May 1996
Ganaxolone	FDA	Treatment of Protocadherin 19 (PCDH19) female epilepsy	24 March 2015	n/a
		Treatment of infantile spasms	25 May 1994	n/a
Lamotrigine	FDA	Treatment of Lennox-Gastaut syndrome	23 August 1995	24 August 1998
Levetiracetam	FDA	Treatment of neonatal seizures	30 April 2010	n/a
Midazolam	FDA	Treatment of nerve agent-induced seizures	24 July 2012	n/a
		Rescue treatment of seizures in patients who require control of intermittent bouts of increased seizure activity (e.g. acute repetitive seizures, seizure clusters)	20 October.2009	n/a
		Treatment of bouts of increased seizure activity in selected refractory patients with epilepsy who are on stable regimens of anti-epileptic drugs and who require intermittent use of midazolam	05 August 2006	n/a
Perampanel	FDA	Treatment of Lennox-Gastaut Syndrome	12 July 2012	n/a
PR-122 (Redox-Phenytoin)	FDA	For the emergency rescue treatment of status epilepticus, grand mal type.	07 May 1990	n/a
PR-320 (Molecusol-Carbamazepine)	FDA	For the emergency rescue treatment of status epilepticus, grand mal type.	20 July 1990	n/a
Pyridoxine (Vitamin B6)	FDA	Treatment of pyridoxine dependent seizures.	3 March 2011	n/a
Repository corticotropin or adrenocorticotropic hormone	FDA	Treatment of infantile spasms	21 May 2003	15 October 2010
Rufinamide	FDA	Treatment of Lennox-Gastaut Syndrome.	10 August 2004	14 November 2008
	EMA	Treatment of Lennox-Gastaut syndrome	20 October 2004	16 January 2007
Stiripentol	FDA	Treatment of Dravet syndrome	30 October 2008	n/a
	EMA	Treatment of Dravet syndrome	5 December 2001	4 January 2007
Sulthiame	FDA	Treatment of patients with benign epilepsy of childhood with centrotemporal spikes (BECTS) also known as rolandic epilepsy	25 July 2013	n/a
Tetracosactide hexaacetate (beta 1-24-corticotropin)	FDA	Treatment of infantile spasms	31 October 2012	n/a
Topiramate	FDA	Treatment of Lennox-Gastaut syndrome	25 November 1992	28 August 2001
Topiramate injection	FDA	Treatment of partial onset or primary generalized tonic-clonic seizures for hospitalized epilepsy patients or epilepsy patients being treated in an emergency care setting who are unable to take oral topiramate	24 July 2013	n/a
Vigabatrin	FDA	Treatment of infantile spasms	6 December 2000	21 August 2009

n/a = not applicable because compound is not approved.

### Pivotal clinical trial designs, primary outcomes, and sponsors

Except for fosphenytoin, where a bioequivalence study in comparison to phenytoin was performed, all other pivotal clinical trials were randomized controlled trials (Table 2). The main outcome measure in most studies was seizure frequency (N = 10 studies). All drug development programs investigating compounds for treatment of West syndrome (N = 2) focused on proportion of seizure-free patients as an outcome measure. Pivotal clinical trials were small (population size between N = 29 and N = 298) and short (12 hours to 19 weeks). For orphan drug designations granted by the FDA, 39 sponsors were pharmaceutical companies and one sponsor was a university (levetiracetam for the treatment of neonatal seizures). For FDA orphan drug designations, 36 sponsors were located in the US, three in the UK, and one in France. For orphan drug designations in the EU, four sponsors were located in the UK, one sponsor in France, and one sponsor in Belgium.

**Table 2:** Pivotal clinical trial design of FDA and EMA approved compounds for rare seizure conditions.

Generic Name	Approved Indication	Study design	N	Age (years)	Study duration	Main outcome measures and top line results*
		Description of pivotal clinical trial				
Clobazam	Adjunctive treatment of seizures associated with Lennox-Gastaut Syndrome inpatients 2 years of age or older	RCT	238	2–54	4 wk baseline, 3 wk titration, 12 wk maintenance period	percent reduction in weekly frequency of drop attacks from 4 wk baseline to 12 wk maintenance period placebo: -12.1% low dose:—41.2% medium dose -49.4% high dose: -68.3%
		add-on therapy with clobazam vs. placebo				
		RCT	68	2–25	4 wk baseline, 3 wk titration, 4 wk maintenance period	percent reduction in weekly frequency of drop attacks from 4 wk baseline to 4 wk maintenance period high dose: -93% low dose: -29%
		high dose vs. low dose				
Diazepam viscous solution for rectal administration	Management of selected, refractory, patients with epilepsy on stable regimens of antiepileptic drugs, who require intermittent use of diazepam to control bouts of increased seizure activity	RCT	91	n/a (47 children, 44 adults)	observation period 12–24 hours after fist application	seizure frequency during the period of observation and a global assessment of severity and nature of seizures diazepam: 0 seizures/hour placebo: 0.3 seizures/hour
		sequential doses of diazepam rectal gel vs. placebo				
		RCT	114	n/a (53 children, 61 adults)	observation period 12 hours after application	seizure frequency during the period of observation diazepam: 0 seizures/12 hours placebo: 2.0 seizures/12 hours
		single doses of diazepam rectal gel vs. placebo				
Felbamate	As adjunctive therapy in the treatment of partial and generalized seizures associated with the Lennox-Gastaut syndrome in children	RCT	n/a	n/a	baseline period, 70 days observation period	seizure frequency during the period of observation, parent/guardian global evaluations felbamate: -26% (total seizures) placebo: +5% (total seizures)
		add-on therapy with felbamate to 1–2 antiepileptic drugs vs. placebo				
Fosphenytoin	For the acute treatment of patients with status epilepticus of the grand mal type	n/a	112	n/a	n/a	infusion tolerance fosphenytoin vs. iv phenytoin local intolerance (9% vs. 90%) infusion disrupted (21% vs. 67%) infusion time (13 min vs. 44 min)
		ADME and infusion tolerance fosphenytoin vs. phenytoin				
Lamotrigine	Adjunctive treatment of Lennox-Gastaut syndrome in pediatric and adult patients.	RCT	169	lamotrigine mean 9,6 years, placebo mean 10.9 years	4 wk baseline, 6 wk titration, 10 wk observation period (2 wk fixed dose, 8 wk dose could be increased)	change in percent from baseline in the frequency of drop attacks and tonic-clonic seizures lamotrigine:-34% (drop attacks) placebo: -9% (drop attacks)
		add-on therapy with Lamotrigine to 1–2 antiepileptic drugs vs. placebo				
Repository corticotropin or adreno-corticotropic hormone	Monotherapy to treat infantile spasms	RCT	29	<2	14 days	number of patients having complete suppression of both clinical spasms and hypsarrhythmia corticotropin: 86.7% prednisone: 28.6%
		high-dose corticotropin vs. prednisone				
Rufinamide	Adjunctive therapy of seizures associated with Lennox-Gastaut syndrome.	RCT	139	4–30	4 wk stable baseline followed by 12 wk double-blind phase (2 wk titration, 10 wk maintenance)	percent change in total seizure frequency per 28 days; The percent change in tonic-atonic (drop attacks) seizure frequency per 28 days; Seizure severity from the Parent/Guardian Global Evaluation of the patient’s condition rufinamide -35.8% (total seizures) placebo -1.6% (total seizures)
		add-on therapy with rufinamide vs. placebo				
Stiripentol	As adjunctive therapy of refractory generalized tonic-clonic seizures in patients with severe myoclonic epilepsy in infancy (Dravet syndrome) whose seizures are not adequately controlled with clobazam and valproate.	RCT	41	>3	4 wk baseline, 8 wk observation	more than 50% reduction in the seizure frequency stiripentol: 72% responders placebo: 5% responders
		add-on therapy to clobazam and valproate				
Topiramate	As adjunctive therapy in patients two years and older with seizures associated with Lennox-Gastaut syndrome	RCT	95	> 2	4 wk baseline, 3 wk titration, 8 wk stabilization	effectiveness were the percent reduction in drop attacks and a parental global rating of seizure severity topiramate: 14.8% (reduction in drop attacks) placebo: -5.1% (reduction in drop attacks)
		add-on therapy with topiramate vs. placebo				
Vigabatrin	For infantile spasms (IS)—1 month to 2 years of age	RCT	221	< 2	Titration over 7 days, followed by a constant dose for 7–14 days.	proportion of patients who were spasm-free for 7 consecutive days beginning within the first 14 days of vigabatrin therapy low-dose: 7% high-dose: 15.9%
		high dose vs. low dose				
		RCT	40	n/a	Baseline 2–3 days, followed by a 5-day treatment phase with vigabatrin or placebo.	post-hoc alternative efficacy analysis (average percent change in daily spasm frequency), using a 24-hour clinical evaluation window: vigabatrin: -68.9% placebo: -17.0%
		vigabatrin vs. placebo				

RCT—randomized controlled trial, n/a—not applicable because complete information is lacking, wk—week or weeks.

*The differences between groups in the pivotal clincial trials listed in top line results were statistically significant. ADME—absorption, distribution, metabolism, elimination

## Discussion

### Productivity output

Our analysis revealed that in the US nine compounds and in the EU two compounds were approved to treat rare seizure conditions. The ratio between orphan drug designation and approval was higher in the EU which is contradictory to general acceptance rates, i.e. 15.4% in the US and 7% in the EU [3]. However, in relation to the low number of designated orphan drugs to treat rare seizure conditions in the EU, the approval of one compound less would comply with an acceptance rate of 7%. Although a common EU/FDA application form is available, which is strongly recommended to facilitate the collaboration between both regulatory authorities, far less orphan drugs are available in the EU. The time advantage of the US Orphan Drug Act may be one reason [3]. However, differences in the reimbursement policies and decisions on prices among Member States in the EU might curtail return of investment and reduce attractiveness of incentives such as marketing exclusivity [23]. One may speculate that the review process in the EU is different from the USA which may account for the observed differences. Additional requirements for qualification as an orphan drug in the EU, such as proofing superiority over existing treatments if any, may compromise European orphan drug applications. However, cannabidiol and fenfluramine hydrochloride were designated as orphan drug in the EU for treatment of Dravet syndrome although stiripentol has been approved seven years earlier for the same condition. According to the public summary of opinion on orphan drug designation, both sponsors apparently have provided sufficient information that Dravet syndrome patients would benefit from both treatments as add on, which needs to be confirmed at the time of marketing authorization [24, 25].

In general, success rates in orphan drug development depend on pivotal clinical trial design (e.g. choice of endpoints and target population), experience of the sponsor, interaction with the legal authorities and disease-specific factors, i.e. the prevalence, disease class, and scientific output [26–28]. By definition, orphan drug development is challenged by small populations. Accordingly, small sample sizes limit clinical trial programs. Therefore, drug development in a more prevalent orphan disease seems to be more favorable [27]. Indeed, most orphan drug approvals were granted for Lennox-Gastaut syndrome which is twice as frequent as West syndrome, six times more frequent than Dravet syndrome and 75 times more frequent than Pyridoxine-dependent epilepsy [4]. In addition, Lennox-Gastaut syndrome is a poly-etiological, age-related epilepsy syndrome with a high disease burden, few treatment options, and thus high medical need for orphan drug development which might facilitate willingness to participate in clinical trials. Sufficient understanding of a disease to identify promising drug targets plays a pivotal role in drug development generating interest from sponsors to initiate a drug development program [27]. Indeed, pathophysiologically well-defined disease such as metabolic diseases, where a missing or dysfunctional enzyme or substrate were identified as the underlying cause, are more likely to result in the development of an orphan drug than orphan diseases with unclear pathomechanisms [27, 28]. In addition, diseases with a high research interest, indicated by a high volume of disease-specific scientific output, such as rare cancers, are more likely to obtain an orphan drug designation than rare diseases with a low number of publications [27, 29, 30]. Current orphan drug legislations may be not sufficient to stimulate successful translation of biomedical research into orphan drug development for diseases with a lower prevalence, such as rare seizure conditions.

### Innovation and efficacy

The US Orphan Drug Act and the EU Orphan Drug Regulation appeared not to substantially stimulate innovative drug development to treat rare seizure conditions which may be in part attributed to lack of basic research. Indeed, mainly compounds that were already approved to treat epilepsy or alternative routes of application for already approved orally available compounds obtained orphan drug designation. In comparison, for rare cancers the majority of compounds were first approved within the US Orphan Drug Act [29]. For example, fosphenytoin was indeed first approved within the orphan drug act but as a pro-drug to the already approved non-orphan phenytoin. Lack of effective and well tolerated antiepileptic orphan drugs is congruent with non-orphan seizure conditions [31]. One important barrier in anticonvulsant drug development may be that drug discovery is mainly relying on two preclinical models, i.e. the maximal electroshock seizure (MES) test for tonic-clonic seizures and the pentylenetetrazole (PTZ) test for generalized nonconculsive seizures. Both tests may fail to discover drugs with novel mechanisms of action for specific orphan epileptic diseases not capturing for example the preclinical efficacy of levetiracetam [31, 32]. The accumulation of cannabidiol orphan drug designations is remarkable. Anecdotal reports suggested an anticonvulsant effect for cannabis extract for many years [33]. However, new interest in this anti-epileptic treatment option increased in 2013, when reports of children benefiting from cannabidiol-enriched cannabis extracts for treatment of pediatric epilepsy appeared [34–36]. Particularly in pediatric epilepsy, intense media interest boosted family members’ belief in the efficacy of cannabidiol [36]. In an open-label uncontrolled trial investigating cannabidiol in intractable epilepsy, the efficacy and safety profile seemed promising [37]. However, the study design entails a strong placebo effect and thus, blinded randomized controlled trials are currently ongoing to evaluate cannabidiol treatment for Dravet syndrome and Lennox-Gastaut syndrome [37]. In general, clinical trial design investigating seizure conditions is challenging because epilepsy per se is a very heterogeneous condition to study and there are no biomarkers or surrogate markers for epilepsy that may be used as an outcome measure. This “neurological trial dilemma” is illustrated in lysosomal storage disorders, a circumscribed group of diseases where the output in drug development is significantly hampered once the study of a condition requires a neurological endpoint [28]. Of interest, while the pivotal clinical trials investigating West syndrome aimed at achieving the probably more difficult clinical endpoint “proportion of seizure-free patients”, most other pivotal clinical trials focused on reducing seizure frequency which is in line with the “EMA Guideline on clinical investigation of medicinal products in the treatment of epileptic disorders” [38] and the “FDA guidelines for the clinical evaluation of antiepileptic drugs” [39]. The numeric output of drugs developed within the US Orphan Drug Act during the last three decades was rather modest for the field of epilepsy (N = 9 approvals), whereas 177 compounds were approved by the FDA for rare cancers, 14 for lysosomal storage diseases, and 14 for rare rheumatologic conditions [28–30].

### Safety aspects

The frequency of safety-related regulatory actions (e.g. safety-related market withdrawals, post-approval black-box warnings, or written communication to healthcare professionals) is lower with orphan drugs than with non-orphan drugs [40]. In addition, clinical trials for the approved compounds were short and small. Small sample sizes may be sufficient for registration trials especially when the size of the effect is large, such as for stiripentol in Dravet syndrome, but this may be an issue in the sensitivity of the orphan drug approach to detect less obvious safety signals. Indeed, the lower number of safety-related regulatory actions could be attributed rather to lower utilization, which is accompanied by lower chances to detect a safety issue, than indicating greater safety for orphan drugs [40]. A close follow-up of treatment outcomes is desirable because antiepileptic drugs in clinical practice are prescribed over years, which is not reflected in the design of clinical registration studies. Orphan drugs approved in an accelerated procedure seem to have an increased risk with regards to safety-related regulatory actions which might counteracted by more intense monitoring as postmarketing obligation and consequently higher chances for detection of a safety issue [40]. As for non-orphan drugs, safety considerations are one possible reason why orphan drug designations are withdrawn before approval [41]. However, the reasons for withdrawal of orphan drug designations for PR-122 Redox-Phenytoin and Pr-320 molecusol-carbamazepine in the US could not be identified. The EMA adopted a negative opinion for midazolam hydrochloride because the sponsor has not established that seizures which continue for at least five minutes affect not more than 5 in 10,000 persons in the Community at the time the application [42].

### Limitations

This analysis exclusively focusses on data from the FDA and EMA. Other jurisdictions in the rest of the world are not included. As the two databases are the largest ones and as the US and EU have ~800 million inhabitants, our results may show a trend in orphan drug development possibly reflecting other drug markets for orphan anti-epileptic compounds. The designation of a compound as an orphan drug was considered a surrogate for the intent to develop a drug for a disease. Not all manufacturers may seek orphan drug designation due to patent considerations. Therefore, the designations submitted to the FDA and EMA may not fully reflect the situation. Time from designation to approval is a somewhat arbitrary measure because it depends at which time point in the development process a sponsor seeks orphan drug designations and thus, a comparison between time-to-approval in the US and EU is not necessarily valid. Although interesting to know, it is not possible to extrapolate how many and which compounds would have been developed without orphan drug legislation.

## Conclusions

Orphan drug development in epilepsy is challenging, no novel molecular entities were developed in the last 30 years under the US Orphan Drug Act. Development focused on already approved antiepileptic or alternative pharmaceutical formulations. In the US more compounds have been approved to treat rare seizure conditions compared to the EU.

## Abbreviations

FDA: Food and Drug Administration
EMA: European Medicines Agency
